# Editorial: Next-generation PROTACs in oncology and beyond: exploring therapeutic targets and their degraders

**DOI:** 10.3389/fphar.2026.1787837

**Published:** 2026-01-29

**Authors:** Daiqing Liao, Debasish Bandyopadhyay

**Affiliations:** 1 Department of Physiology and Aging, College of Medicine, and UF Health Cancer Institute, University of Florida, Gainesville, FL, United States; 2 School of Integrative Biological and Chemical Sciences (SIBCS), University of Texas Rio Grande Valley, Edinburg, TX, United States

**Keywords:** breast cancer, cancer immuno therapy, inflammation, peptide therapeutics, proteolysis-targeting chimeras (PROTACs), targeted protein degradation

Proteolysis-targeting chimeras (PROTACs) are synthetic heterobifunctional molecules that combine a ligand for a protein-of-interest (POI) and one for an E3 ubiquitin ligase with a short chemical linker. Such molecules induce polyubiquitination of the POI and subsequent degradation by the proteasome. 2026 marks a quarter-century of the PROTAC field, progressing from a transformative innovation to the cusp of clinical application. This journey began in 2001 when the laboratories of Craig Crews and Raymond Deshaies reported the first PROTAC (Protac-1) consisting of a covalent POI-binder linked to a peptide-based E3 ligase recruiter. Protac-1, while impractical as pharmaceuticals due to issues related to cell permeability and stability, led to a critical turning point in 2015 when potent PROTACs using small-molecule ligands for the E3 ligases Cereblon (CRBN) and von Hippel-Lindau protein (VHL) were discovered. These advancements transformed PROTACs from niche chemical biology tools into a promising therapeutic modality.

Within the broader field of induced-proximity pharmacology, several other modalities such as molecular glue degraders (MGDs) progress steadily along with the more mature but still actively expanding PROTAC technology. A recent breakthrough—the first major Phase 3 clinical success of the estrogen receptor (ER)-targeting PROTAC vepdegestant (ARV-471) in hormone receptor–positive metastatic breast cancer—further validates the potential of targeted protein degradation. Vepdegestant improves survival outcomes compared with the standard-of-care agent fulvestrant in patients harboring ER mutations. Fulvestrant, a selective ER degrader (SERD), promotes ER degradation by disrupting ER folding, a mechanism distinct from PROTAC-mediated degradation. These mechanistic differences underscore the unique clinical value of PROTACs in overcoming resistance to existing therapies. Furthermore, PROTACs can eliminate disease-driving proteins previously considered undruggable, including multi-domain, intrinsically disordered, and scaffolding proteins.

Celastrol (also known as tripterine) is a natural pentacyclic triterpene isolated from the root bark of the Thunder God Vine (*Tripterygium wilfordii*, or *Lei Gong Teng*), long used in traditional Chinese medicine for treating inflammatory diseases. Beyond its anti-inflammatory properties, celastrol exhibits antitumor activity through suppression of oncogenic signaling pathways and engagement of multiple intracellular targets in cancer cells. In the article by Gu et al. the authors report a novel PROTAC, YX-112, generated by linking celastrol to the CRBN-recruiting ligand thalidomide *via* a rigid linker. Proteomic profiling identified various potential targets, among which CHEK1, a kinase essential for DNA damage–induced cell-cycle arrest and DNA repair, was validated by Western blot analysis. Notably, YX-112 demonstrated enhanced anti-proliferative activity against triple-negative breast cancer cells compared with celastrol.

Peptides have emerged as a valuable source of novel therapeutics due to advantages such as reduced toxicity, high specificity, and lower development costs. Because many protein–protein interactions are mediated by short peptide motifs from one binding partner engaging a structured domain of another, these peptides have been increasingly explored as ligands for PROTAC design. In this Research Topic, a review article by Li et al. highlights recent advances in peptide-based PROTACs.

In the review article titled “Indoleamine 2,3-dioxygenase 1 in cancer immunotherapy: from small-molecule inhibition to PROTAC-mediated degradation” Li et al. discussed the role of Indoleamine 2,3-dioxygenase 1 (IDO1) in cancer immunotherapy and its interactions with small molecule inhibitors and PROTAC-mediated degraders. The article reviews IDO1 as a central immunometabolic checkpoint that tumors exploit by converting tryptophan to kynurenine, thereby depleting tryptophan for effector T cells and activating aryl hydrocarbon receptor–driven regulatory pathways. It summarizes that first-generation small-molecule IDO1 inhibitors, although biologically rational and effective in preclinical models, showed inconsistent, often disappointing results in late-stage clinical trials, including the ECHO-301/KEYNOTE-252 study, highlighting limitations of simple enzymatic blockade and challenges in patient selection. Against this backdrop, the authors introduce PROTACs as an event-driven strategy that recruits IDO1 to E3 ubiquitin ligase-mediated degradation, thereby simultaneously removing both catalytic and non-enzymatic functions. The review details emerging IDO1-directed PROTACs and nano-PROTAC platforms, emphasizing durable target knockdown, the capacity to remodel the tumor microenvironment, and the potential to synergize with immune checkpoint inhibitors, and concludes that optimized degraders, plus biomarker-guided stratification, may revitalize IDO1-targeted cancer immunotherapy.

Mixed lineage kinase domain-like protein (MLKL) traditionally executes necroptosis by oligomerizing upon RIPK3 phosphorylation and disrupting plasma membranes, but recent insights reveal its broader roles in modulating organelle dynamics across mitochondria, lysosomes, endosomes, endoplasmic reticulum (ER), and nucleus. In the review title “MLKL as an emerging machinery for modulating organelle dynamics: regulatory mechanisms, pathophysiological significance, and targeted therapeutics” Wang et al. elaborate on the structural features of MLKL that underlie its function and are crucial for drug discovery. Further, they have summarized the regulation of MLKL across various cellular organelles, including mitochondria, lysosomes, endosomes, endoplasmic reticulum, and the nucleus. Also, they have drawn the interaction network of components involved in MLKL’s actions across these diverse organelles.

This special Research Topic *in Frontiers in Pharmacology*, “Next-Generation PROTACs in Oncology and Beyond,” aims to explore current advances of the PROTAC field and brings together research that pushes the boundaries of targeted protein degradation for our broad readership.

